# Research on the Method for Recognizing Bulk Grain-Loading Status Based on LiDAR

**DOI:** 10.3390/s24165105

**Published:** 2024-08-06

**Authors:** Jiazun Hu, Xin Wen, Yunbo Liu, Haonan Hu, Hui Zhang

**Affiliations:** School of Intelligent Systems Engineering, Sun Yat-sen University, Shenzhen 518107, China; hujz@mail2.sysu.edu.cn (J.H.);

**Keywords:** LiDAR, 3D point cloud, bulk grain loading, point cloud classification, point cloud segmentation

## Abstract

Grain is a common bulk cargo. To ensure optimal utilization of transportation space and prevent overflow accidents, it is necessary to observe the grain’s shape and determine the loading status during the loading process. Traditional methods often rely on manual judgment, which results in high labor intensity, poor safety, and low loading efficiency. Therefore, this paper proposes a method for recognizing the bulk grain-loading status based on Light Detection and Ranging (LiDAR). This method uses LiDAR to obtain point cloud data and constructs a deep learning network to perform target recognition and component segmentation on loading vehicles, extract vehicle positions and grain shapes, and recognize and make known the bulk grain-loading status. Based on the measured point cloud data of bulk grain loading, in the point cloud-classification task, the overall accuracy is 97.9% and the mean accuracy is 98.1%. In the vehicle component-segmentation task, the overall accuracy is 99.1% and the Mean Intersection over Union is 96.6%. The results indicate that the method has reliable performance in the research tasks of extracting vehicle positions, detecting grain shapes, and recognizing loading status.

## 1. Introduction

**Figure 1:**
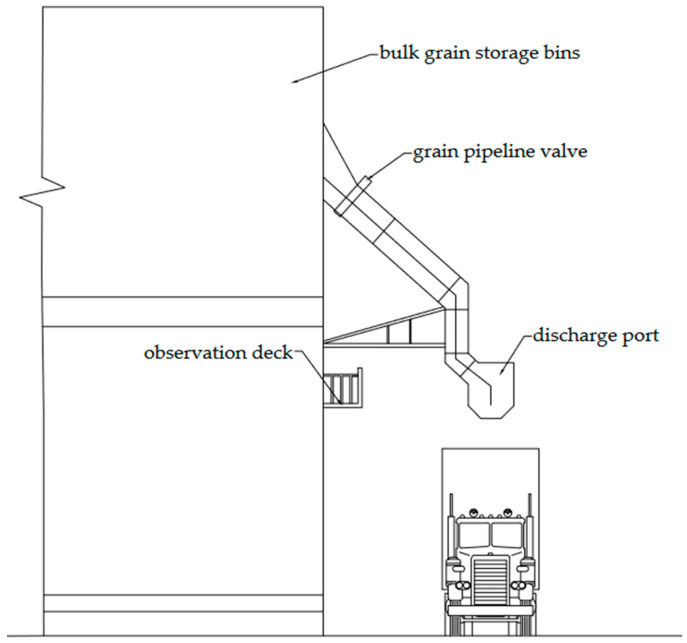
Schematic diagram of bulk grain-loading site.

Grain is a common type of cargo for transportation. Due to its fluidity, bulk transportation of grain can better utilize the space of transport vehicles and improve loading and unloading efficiency. Therefore, in practice, grain is usually transported in bulk. The loading site for bulk grain cargo is shown in Figure 1. The grain storage bins open the grain pipeline valve, using gravity to directly pour the grain from the discharge port into the transport vehicle. When the outflowing grain reaches the set weight, the valve is closed to stop the discharge port. In this process, to fully utilize the vehicle’s space and prevent the grain from overflowing excessively, the loading workers must climb to a high position to observe the loading situation inside the vehicle and notify the driver to adjust the vehicle’s position. However, due to the characteristics of bulk grain, a large amount of dust is inevitably generated at the loading site, and the loading equipment produces noise during operation. This makes it difficult for loading workers to observe the grain’s condition inside the vehicle and communicate with the driver using traditional methods. This process results in high labor intensity for the loading workers, significant safety hazards over long-term work, and low grain-loading efficiency. Therefore, it is worth researching and exploring how to automatically guide the driver to adjust the vehicle’s position, reduce the labor intensity of the loading workers, eliminate safety hazards during the loading process, and ultimately improve overall loading efficiency.

In recent years, with the development of deep learning and perception technologies, sensors have been widely applied in various important fields such as transportation, logistics, robotics, and industrial production [1,2,3]. Using sensors to obtain scene information, and performing target recognition or semantic segmentation based on perceptual data, has become the key to achieving automation and intelligence [4,5]. To address the aforementioned issues, suitable sensors can be selected to acquire bulk grain-loading information, extract the vehicle’s position and the grain’s shape inside the vehicle, recognize the bulk grain-loading status, guide the vehicle to adjust its position based on the loading status, and achieve automated grain loading.

In practical engineering, bulk grain-loading tasks need to be carried out according to production schedules, with timing that is not fixed but is influenced by weather conditions. At the loading site when there are no loading tasks, staff or working vehicles will also pass through the loading area. Their main modes of passage include walking, cycling, and driving. Obviously, these unrelated individuals must not affect or interfere with the recognition of the bulk grain-loading status. To recognize the bulk grain-loading status, it is necessary to identify the vehicle that is to be loaded, determine the vehicle’s position, and perceive the shape of the grain and the vehicle’s information. Cameras find it difficult to obtain precise depth information and are greatly affected by lighting conditions. If cameras are used for perception, it is challenging to accurately capture vehicle and grain data during the loading process, and additional lighting equipment is needed for nighttime operations. LiDAR, on the other hand, can directly generate three-dimensional point cloud data with high measurement accuracy and robustness, and it is suitable for long-distance and nighttime work. This makes LiDAR applicable in many environments where cameras struggle to function effectively [6]. The three-dimensional point cloud data from bulk grain loading, which includes precise vehicle position information and grain shape data, forms the basis for recognizing the loading status.

Deep learning technology, as an efficient representation learning algorithm, can extract key features from these point cloud data to perform complex object-detection and -segmentation tasks [7,8]. PointNet [9] has set a milestone in the deep learning processing of point clouds, directly extracting global features from point sets, but it has encountered difficulties in capturing local geometric details. PointNet++ [10] introduces a hierarchical network structure that can capture hierarchical features from local to global, significantly improving the processing capability for complex point cloud data and fine local structures. Building on this, PointNeXt [11] has further enhanced performance and efficiency by improving training strategies, introducing separable MLP (Multilayer Perceptron), and incorporating inverted residual MLP. These advancements allow the network to learn more complex features and representations from point cloud data, leading to better generalization and robustness in various point cloud-processing tasks. DGCNN [12] uses a graph convolutional network to process point clouds, effectively capturing local features through a dynamic graph structure, and LDGCNN [13] optimizes the network structure by connecting hierarchical features of different dynamic graphs, effectively addressing the gradient vanishing problem. Shellnet [14], with its statistical data from concentric spherical shells, effectively resolves the issue of order ambiguity in point cloud data, maintaining high performance with a relatively small number of model layers. PointMLP [15] introduces a pure residual network equipped with lightweight geometric affine modules, significantly enhancing the speed and accuracy of point cloud processing.

Although the above methods can all accomplish point cloud-classification and -segmentation tasks, their different processing methods mean that their application scenarios also vary. Convolutional neural networks processing point clouds are often used for identifying and separating complex structures [16]. They are widely applied in semantic segmentation tasks in urban road areas, 3D mapping fields, and medical fields [17,18,19]. Directly processing point clouds is more computationally efficient and more suitable for object detection and segmentation in the automation field [20], such as recognizing human actions [21], segmenting object parts, and measuring target volumes [22]. The bulk grain-loading process is a fixed scene for identifying and segmenting specific targets, where the collected point cloud data types are relatively few, and there is no need for overly complex model structures to recognize multiple types of targets. As a classic network that directly processes point clouds, PointNet++ is more suitable for the grain-loading task due to its lower parameter count and faster training speed. However, the point clouds in the bulk grain-loading process have characteristics such as feature loss due to grain or dust occlusion, a large number of points, complex processing, and sparse points at intersections and edges. Therefore, we propose a novel method tailored for the bulk grain-loading process, which is based on the PointNet++ network and adjusted to address the unique challenges of this application by incorporating specific characteristics of bulk grain loading.

Based on the above context, this paper conducts research on the method for recognizing the bulk grain-loading status based on LiDAR. Using dual LiDAR sensors for joint perception ensures complete coverage of the loading area. Data preprocessing is carried out to eliminate interference points and fill in occluded parts. A bulk grain-loading status-recognition network, PNGL (Point Net Grain Loading), is constructed to classify the target point cloud, extract high-dimensional features of the vehicle, and achieve vehicle target detection, thereby avoiding missed and false detections. The vehicle components are finely segmented to obtain vehicle information and grain shape, and the grain-loading status is determined. The current status and prompt information are output to assist operators in loading and unloading tasks, promoting the automation of bulk grain loading. This research effectively addresses the shortcomings of traditional judgment methods, reduces the labor intensity of operators, eliminates safety hazards, improves the efficiency of bulk grain loading, and has significant importance for achieving automated loading.

## 2. Overview

### 2.1. LiDAR Detection Principle

LiDAR is a sensor that measures distance and generates three-dimensional point clouds by emitting laser beams and detecting their reflections. It calculates distance by recording the time of flight or phase shift of the laser pulses, thereby obtaining the three-dimensional coordinates of target objects. Among these methods, the time-of-flight (ToF) method is widely used in industrial applications such as long-distance measurement, autonomous driving, and drone mapping due to its wide measurement range, strong applicability, and intuitive results [23]. The distance *h* between the LiDAR and the target object is shown in Equation (1).

(1) $$h=\frac{c\times \Delta t}{2}$$

where Δ*t* is the time gap between the transmitted laser and the received laser, and *c* is the speed of light.

To ensure data accuracy and perception effectiveness, considering factors such as LiDAR detection range, scanning frequency, field of view, and cost, this paper selects a sixteen-line mechanical LiDAR, The “16-line” indicates that the LiDAR has 16 laser receiver modules, while “mechanical” refers to the sensor’s scanning method [24]. The specific parameters are shown in Table 1.

### 2.2. Problem Overview

Based on field investigations, this research needs to address the following problems:

- Limited Perception Range: The presence of large loading and unloading equipment and components on the vehicle body causes occlusions for the LiDAR sensors at the loading site.
- Interference from Irrelevant Points: Due to the characteristics of grain, the loading site often has dust, which interferes with feature extraction. Additionally, this dust can obscure parts of the target vehicle, leading to missing point cloud data.
- False detections and missed detections: During the loading task, it is necessary to detect the target vehicle, determine its position, and extract the grain-loading shape to avoid missed detections. When the loading task is not being performed, the perception area should also accommodate other engineering vehicles and personnel. In such cases, the method should remain unaffected and avoid false detections.
- Loading Assistance: The driver needs to dynamically adjust the vehicle position based on the loading status to make full use of the cargo area space and avoid grain overflow accidents.

By addressing these issues, the research aims to develop a robust method for recognizing the bulk grain-loading status, thereby enhancing the automation and efficiency of the grain-loading process.

### 2.3. Methods

In summary, to address the above-mentioned problems, this paper proposes the following handling methods:

- Installation of Two Multi-line LiDAR Sensors: To broaden the perception range and eliminate the impact of occlusions. These sensors complement each other to provide a wider perception range and reduce the effects of occlusions.
- Preprocessing of Raw Data: To ensure the integrity and accuracy of the point cloud data. This ensures that the key features of the vehicle and grain point clouds are maintained.
- Classification of Point Clouds: To accurately identify the target vehicle and prevent false detections and missed detections. This achieves accurate target vehicle identification and prevents false detections and missed detections.
- Segmentation of Vehicle Components: To determine the vehicle position and extract grain point clouds. Segment the point cloud of vehicle components and use the relationships between components to determine the vehicle’s position. Extract the grain point clouds and recognize the loading status based on the point cloud shape.
- Output Prompts Based on Loading Status: To guide the vehicle in completing the loading task and improve grain-loading efficiency.

**Figure 2:**
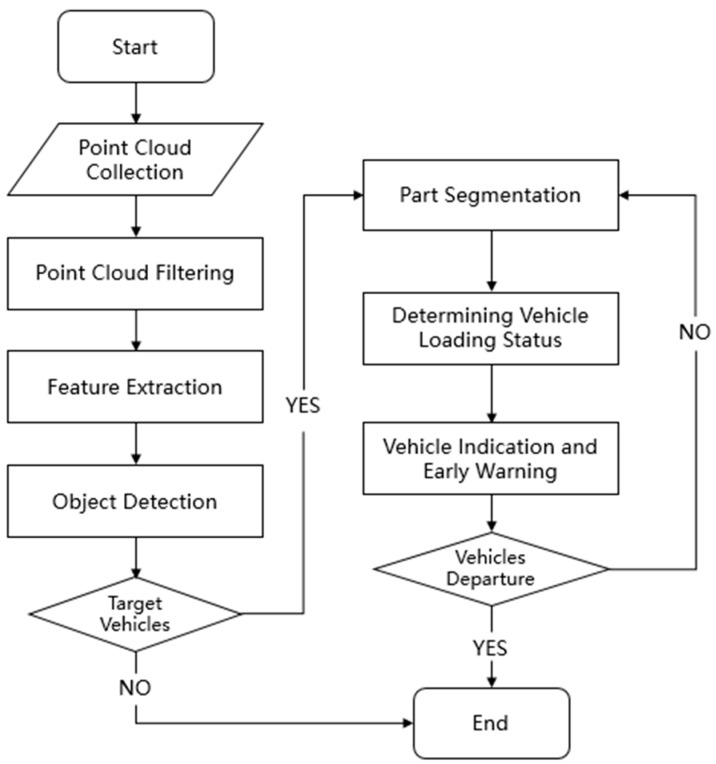
Flowchart of bulk grain-loading status recognition.

The proposed flowchart for recognizing the bulk grain-loading status is shown in Figure 2.

## 3. Method for Recognizing Bulk Grain-Loading Status

### 3.1. Sensor Installation

**Figure 3:**
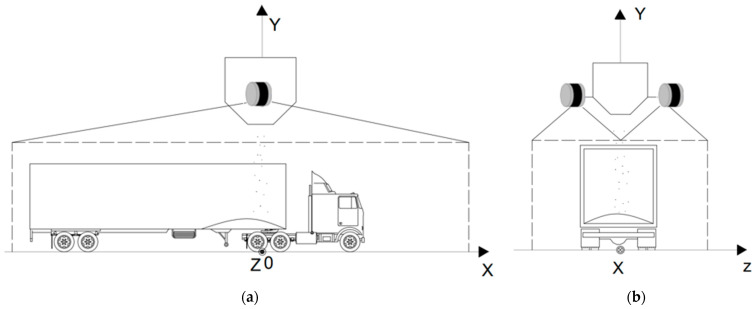
(**a**) The front view of the sensor installation locations; (**b**) the side view of the sensor installation locations.

To solve the problem of limited perception during bulk loading, it is necessary to reasonably plan the installation positions of the sensors. The principle is to ensure that the sensors are easy to install and reproducible without affecting their performance. The sensor installation positions in this paper are shown in Figure 3. Specifically, LiDAR sensors are installed on both sides of the discharge port, 30 cm away from it, and a perception coordinate system is established. The origin is the ground directly below the discharge port, with the vehicle’s forward direction as the *x*-axis, the vertical upward direction as the *y*-axis, and the left-to-right direction as the *z*-axis.

As shown in Figure 3, increasing the installation height of the LiDAR sensors to expand the perception range would cause significant occlusion of the LiDAR’s field of view by the discharge port. Using higher line beam and larger range LiDAR sensors would lead to incomplete perception due to occlusion by the vehicle’s walls. Based on the above analysis, we chose to install the LiDAR sensors at the fixed positions shown in Figure 3 to collect point cloud data of the vehicles and grain in the loading area.

### 3.2. Sensor Calibration and Fusion

**Figure 4:**
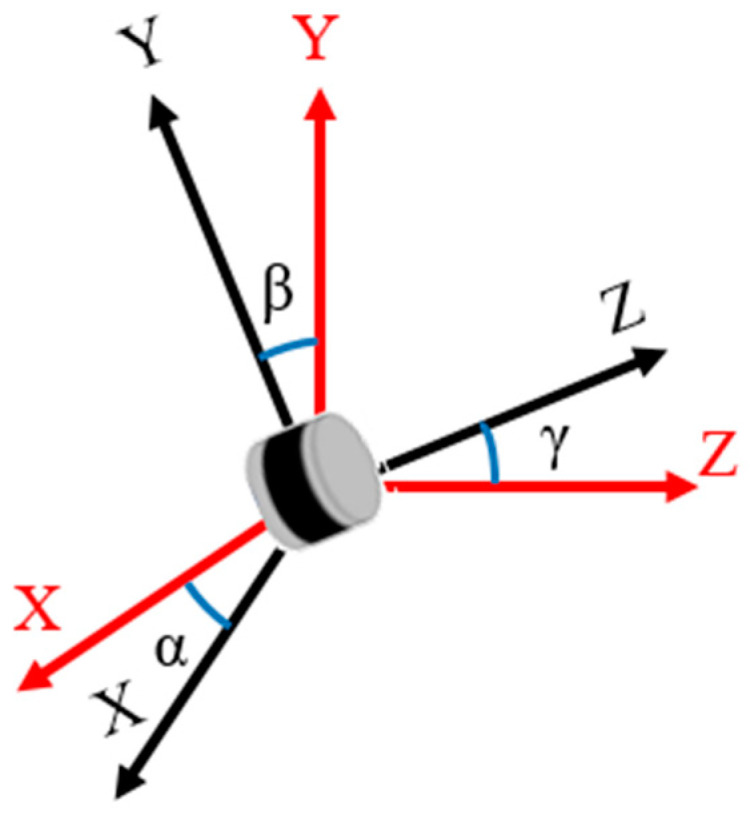
LiDAR coordinate system. The black coordinates represent the coordinate system before calibration, while the red coordinates represent the coordinate system after calibration.

During the installation of LiDAR sensors, variations in fixed components and installation methods can lead to slight tilts in the LiDAR, as shown in Figure 4. The tilt angle can cause discrepancies in the actual position of targets, affecting subsequent recognition and judgment. To ensure high-quality point cloud data collection and achieve LiDAR data synchronization, it is necessary to calibrate the LiDAR sensors to eliminate tilt angle errors.

For LiDAR calibration, this study references Wen’s method [25], utilizing an adaptive approach for point cloud calibration. The advantage of this method is that it maintains an error within 0.1 degrees, does not require a calibration board, and is more suitable for the installation and operation of multiple devices in industrial scenarios. The specific method is as follows: Perceive the loading area without any vehicles to obtain a dense ground point cloud. Use the Density-Based Spatial Clustering of Applications with Noise (DBSCAN) algorithm to fit and separate the complete ground point cloud. Calculate the angle between the ground point cloud and the ideal coordinate system. Transform the original point cloud using the corresponding rotation and translation matrices in three directions, as shown in Equation (2), to obtain the point cloud data in the target coordinate system.

(2) $$\left[\begin{array}{l} x^{\prime }\\ y^{\prime }\\ z^{\prime } \end{array}\right]=\left[\begin{array}{ccc} 1 & 0 & 0\\ 0 & \cos\alpha & -\sin\alpha \\ 0 & \sin\alpha & \cos\alpha \end{array}\right]\times \left[\begin{array}{ccc} \cos\beta & 0 & -\sin\beta \\ 0 & 1 & 0\\ \sin\beta & 0 & \cos\beta \end{array}\right]\times \left[\begin{array}{ccc} \cos\gamma & -\sin\gamma & 0\\ \sin\gamma & \cos\gamma & 0\\ 0 & 0 & 1 \end{array}\right]\times \left[\begin{array}{l} x_{0}\\ y_{0}\\ z_{0} \end{array}\right]$$

where *α* is the tilt angle in the *x*-axis direction, *β* is the tilt angle in the *y*-axis direction, and *γ* is the tilt angle in the *z*-axis direction.

**Figure 5:**
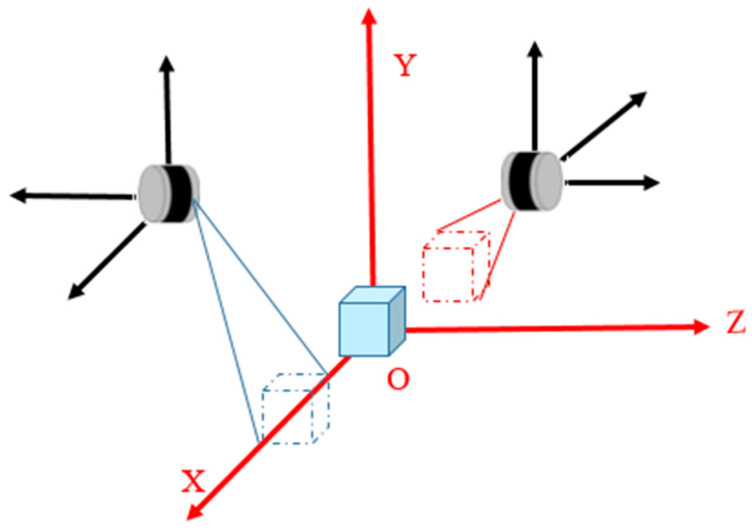
Dual LiDAR data fusion. The black coordinate system represents the LiDAR coordinate system, and the red coordinate system represents the loading site coordinate system. The solid cube indicates the real target, while the dashed cube represents the target point cloud.

As shown in Figure 5, after calibration, the point clouds obtained by the two LiDARs do not match the target point cloud in the loading site coordinate system. To ensure that the point cloud matches the real target, it is necessary to perform translation and rotation transformations on the target point cloud to achieve data fusion.

For the translation transformation, since the LiDAR positions are fixed, the translation vector in the three directions can be calculated by directly measuring the difference between the target point cloud and the actual position of the target.

For the rotation transformation, since the LiDARs have already been calibrated, it is only necessary to convert the LiDAR coordinate system to the loading site coordinate system. Specifically: For the left LiDAR, reverse its *z*-axis, with the rotation vector being. For the right LiDAR, reverse its *x*-axis, with the rotation vector being. By applying these transformations, the coordinate systems can be unified and data fusion can be achieved.

### 3.3. Data Preprocessing

Point cloud data are subject to noise, duplicate points, and outliers due to factors such as the sensor’s installation angle, detection range, and dust interference. If left unprocessed, these issues can adversely affect recognition performance. To eliminate irrelevant points and enhance system performance, this study employs pass-through filtering and statistical filtering to remove interference points.

Pass-through filtering is often used for initial data processing, allowing for the direct extraction of point cloud data within a specified range. After pass-through filtering, the point cloud coordinates within a length of 25 m, a width of 6 m, and a height of 6 m are obtained.

**Figure 6:**
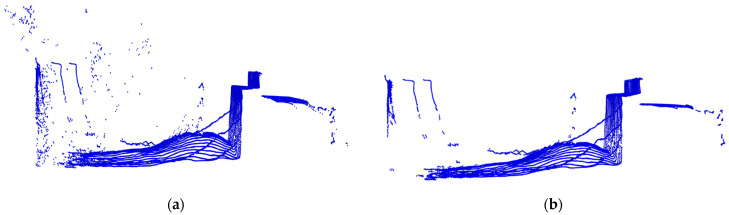
(**a**) Before point cloud filtering; (**b**) after point cloud filtering.

Statistical filtering can effectively remove outliers in the point cloud, eliminating the interference caused by dust during the bulk grain-loading process and ensuring the effectiveness of model training. The specific process is shown in Equation (3), where *μ* represents the average distance from a certain point to all other points, *n* is the total number of points, *σ* denotes the standard deviation of the distances, and *S* indicates a custom standard deviation coefficient. Using a distance threshold *D_max_* as a reference the average distance between each point and its 8 nearest neighbors is calculated. If this distance exceeds the threshold, the point is considered an outlier. In this study, through multiple experiments, the parameters have been set such that the number of nearest neighbors is 8, and *S* is set to 1 for statistical filtering. The results are shown in Figure 6.

(3) $$D_{\max}=\mu +S\times \sigma ,$$

(4) $$\mu =\frac{1}{n}\sum_{i=1}^{n}D_{i},$$

(5) $$\sigma =\sqrt{\frac{1}{n}\sum_{i=1}^{n}\left(D_{i}-\mu \right)}$$

### 3.4. Dataset Construction

This study conducted field data collection at a grain terminal in a port located in southeastern China. The primary types of grain at this terminal include wheat, corn, and soybean meal. The terminal has nearly one hundred grain-loading and -unloading platforms, with an annual throughput exceeding 20 million tons, highlighting a significant and urgent need for automated grain handling.

The main vehicle types used for grain transport at this port are large trailers and medium-sized trucks. When there are no loading tasks, the bulk grain-loading area is also used for vehicle and pedestrian passage, where pedestrians can move either on foot or by bicycle. Two LiDAR sensors were installed at the positions shown in Figure 3, and point cloud data of vehicles and pedestrians passing through the monitored area were collected multiple times. In total, 3195 samples of vehicles and pedestrians under various conditions were gathered.

Subsequently, the collected point cloud data were manually classified and labeled to form a bulk grain-loading dataset. To minimize discrepancies due to different classification standards and segmentation boundaries among different individuals, the labeling was conducted by the same person using the open-source software CloudCompare V2.12.4.

**Figure 7:**
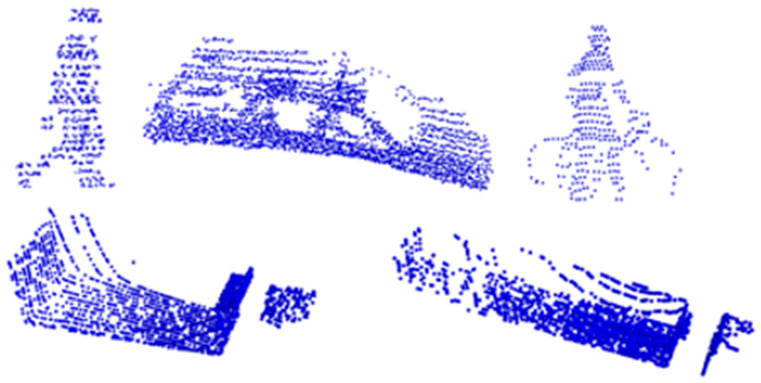
Five categories of targets. From left to right and top to bottom, the targets are pedestrians, cars, bicycles, trucks, and trailers.

**Figure 8:**
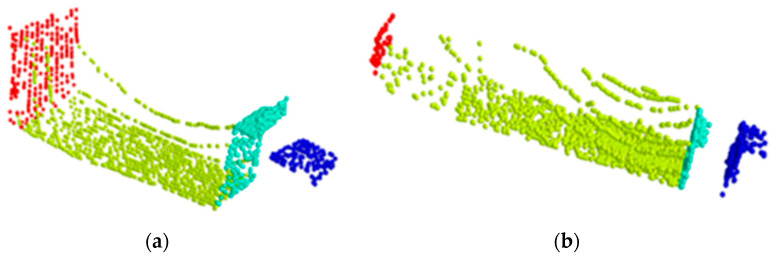
Labeled body parts, where blue is cab of vehicle, cyan is front wall of cargo area, green is cargo area, and red is rear wall of cargo area. (**a**) Truck. (**b**) Trailer.

After processing the collected data through fusion, classification, and cleaning, a total of 2810 samples were obtained for the point cloud-classification dataset and the component-segmentation dataset. The point cloud-classification dataset was divided into five categories: pedestrians, cars, bicycles, trucks, and trailers. The results are shown in Figure 7, where the point clouds have been normalized and centered to ensure visualization clarity. The segmentation dataset includes two categories: trucks and trailers, with each category further divided into eight components: truck cab, front wall of the truck body, interior of the truck body, rear wall of the truck body, trailer cab, front wall of the trailer body, interior of the trailer body, and rear wall of the trailer body. The manually labeled components of the vehicles are shown in Figure 8, and the data for each part are listed in Table 2.

### 3.5. PNGL Network Design

Based on the collected data, it is observed that the bulk grain-loading point cloud has certain characteristics such as partial occlusion, a large number of points, dust interference when grain is falling, and sparse edges of the vehicle body. To accommodate these characteristics, we propose an improved PointNet++ network named PNGL (Point Net Grain Loading).

The PNGL network adopts octree sampling, which provides faster processing speeds and better preservation of spatial features of the point cloud, thereby reducing dust interference. Additionally, since the point clouds of different vehicle types and different batches of loading are quite similar, and the vehicle point clouds are mobile during the loading process, the network employs global average pooling to reduce the risk of overfitting, decrease sensitivity to positional information, and extract global features.

**Figure 9:**
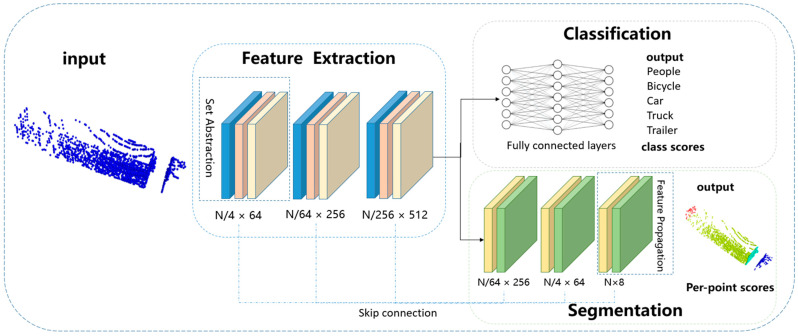
PNGL network.

The structure of the PNGL network is shown in Figure 9. It consists of three main parts: feature extraction, point cloud classification, and component segmentation.

**Figure 10:**
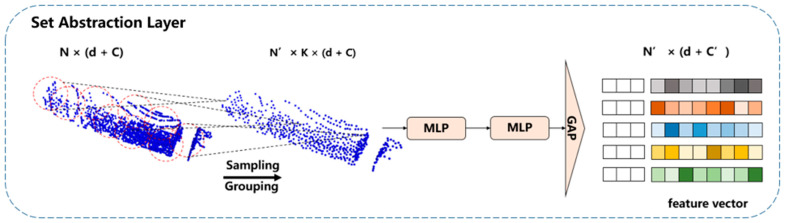
Set abstraction layer, where *N* and *N*′ are the numbers of point clouds before and after sampling, respectively, *K* represents the number of neighboring points, *d* denotes coordinate information, *C* is the number of features, *C*′ is the number of newly generated features, and GAP stands for global average pooling.

**Figure 11:**
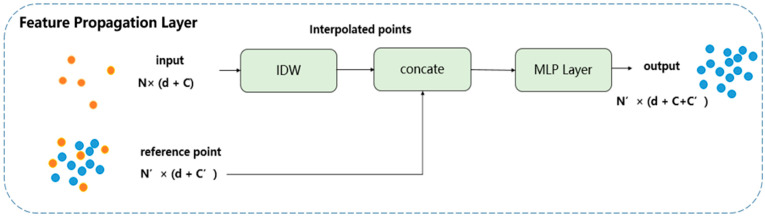
Feature propagation layer, where IDW stands for inverse distance weighted.

- Feature Extraction: This part includes three set abstraction layers to recursively extract multi-scale features at the scale of {1/4, 1/64, 1/256} for the input point cloud consisting of *N* points. The raw point cloud data is processed through these layers, transforming it into high-dimensional nonlinear representations and aggregating features within both local and global regions. Specifically, the first set abstraction layers take an *N* × 3 matrix as the input and outputs an N/4 × 64 matrix of N/4 subsampled points with 64 dimensional feature vectors summarizing the local contextual information. Following the same principle, the second and third set abstraction layers proceed in the same manner, ultimately yielding an N/256 × 512 matrix. Figure 10 shows an example set abstraction layers.

- Point Cloud Classification: The extracted features are input into fully connected layers to output the corresponding categories. To prevent overfitting and increase the model’s generalization ability, dropout is introduced in the fully connected layers for random drop processing.
- Component Segmentation: This part consists of three feature propagation layers. These layers perform inverse distance interpolation and concatenation on the extracted high-dimensional features to form new features. Through progressive upsample, each point obtains sufficient contextual information during segmentation, thus improving segmentation accuracy. The first feature propagation layer output data size of the encoder is N/64 × 256 (where 256 is the dimension of features). Similarly, the second and third feature propagation layers receive input from the preceding layer. Ultimately, the target point cloud is segmented into 8 parts. Figure 11 shows an example of the feature propagation layers.

In summary, the PNGL network is specifically designed to handle the unique challenges presented by bulk grain-loading point clouds, including occlusions, large datasets, dust interference, and sparse edges. By employing octree sampling and global average pooling, along with set abstraction and feature propagation layers, the PNGL network effectively extracts and processes high-dimensional features to achieve accurate point cloud classification and component segmentation.

The set abstraction layer consists of three sub-layers: the Sampling Layer, the Grouping Layer, and the PointNet Layer. As shown in Figure 10, the process is as follows:

- Sampling Layer: The point cloud data is input, and octree sampling selects a set of sampled points *N*′. These points define the centroids of local regions.
- Grouping Layer: Using a query ball, this layer constructs local region sets by finding *K* points within the radius of a sphere around each centroid. The output point set at this stage is *N*′ *× k ×* (*d* + *C*), where *K* represents the number of points in the neighborhood of each centroid point. Each set abstraction module has different values for the sampling number *K* and the sampling radius *R*. Additionally, *K* and *R* increase with each layer, allowing the set abstraction to capture local point cloud features at different scales. Multiple set abstractions output the global features of the point cloud.
- PointNet Layer: This layer uses a small PointNet network to encode the local region patterns into feature vectors. It employs a multi-layer perceptron (MLP) structure and applies global average pooling to the features. The resulting point set after feature extraction by the PointNet layer is *N*′ *×* (*d* + *C*′).

The detailed operations within each sub-layer are as follows: Sampling Layer: Selects a subset of points from the input point cloud using octree sampling, which efficiently captures the structure and density variations in the point cloud. Grouping Layer: For each sampled point (centroid), this layer groups neighboring points within a certain radius to form local regions. The radius and the number of neighbors increase with each subsequent layer, allowing the network to capture features at varying scales. PointNet Layer: Each local region is passed through a small PointNet network. This involves applying shared MLPs to each point in the local region, followed by max pooling to obtain a feature vector that represents the entire region. Global average pooling is then applied to aggregate these features, resulting in a compact representation of the point cloud’s global features. 

By combining these three sub-layers, the set abstraction layer effectively captures and encodes local and global features of the point cloud. This multi-scale feature-extraction process is crucial for accurately classifying and segmenting point clouds in the context of bulk grain loading, where point clouds are characterized by occlusions, dust interference, and varying densities.

For the component-segmentation task, the network needs to progressively propagate the extracted high-dimensional features back to the original point set to ultimately obtain the class label for each point. This method decodes the features through a three-layer feature propagation mechanism, as shown in Figure 11. 

The process of feature propagation is as follows: To propagate the point features from *N ×* (*d* + *C*) to *N*′ this study uses inverse distance weighted interpolation [10] (IDW) as shown in Equation (6), where *p* is set to 2 and *m* is set to 3. The feature values at the coordinates of *N*′ are interpolated from the features of *N* to achieve feature propagation. These interpolated features are then concatenated with the previous layer’s features and passed through a MLP with shared fully connected layers and activation functions to update the feature vector of each point. This process is repeated until the features are propagated to the original point set, ultimately achieving point cloud segmentation.

(6) $$f^{\left(j\right)}(x)=\frac{\sum_{i=1}^{m}w_{i}(x)f_{i}^{\left(j\right)}}{\sum_{i=1}^{m}w_{i}(x)},$$

(7) $$w_{i}\left(x\right)=\frac{1}{d{\left(x,x_{i}\right)}^{p}}$$

where *f* is the eigenvalue, *p* denotes the degree of influence of distance on weights, and *m* denotes the interpolation calculation by taking *m* points in the known point set.

### 3.6. Recognition of Loading Status

**Figure 12:**
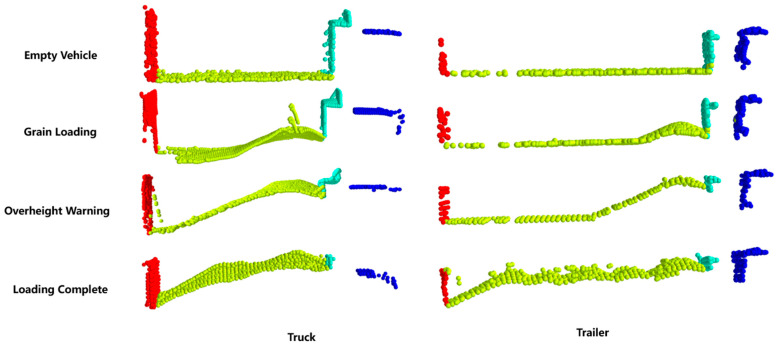
Bulk grain-loading status. Blue represents the vehicle’s cab, cyan represents the front wall of the cargo area, green represents the cargo area itself, and red represents the rear wall of the cargo area.

The aforementioned model is applied to the bulk grain-loading process for the classification and segmentation of target vehicles. By calculating the maximum, minimum, and average coordinates of the point clouds of each segmented part, vehicle data can be obtained. The loading status of the bulk grain can then be determined based on the acquired vehicle data, as shown in Figure 12. Currently, the loading status are divided into the following five categories:

- Empty Vehicle: The average height of the compartment is close to the height from the bottom of the compartment to the ground, approximately the minimum value.
- Grain Loading: The height of the point cloud in the compartment below the discharge port gradually increases.
- Overheight Warning: The height of the point cloud in the compartment below the discharge port is about to exceed the height of the front wall of the compartment.
- Loading Complete: The average height inside the compartment is close to the height of the compartment walls, and the rear wall of the compartment is near the discharge port.
- Standby: There is no target vehicle in the loading area, and the system is in standby mode.

Based on the status outputs, the following instructions are executed:

- Empty Vehicle: Calculate the vehicle’s relative position and direct the vehicle to move so that its front wall slightly exceeds the discharge port.
- Grain Loading: Calculate the grain height in the discharge port area and the grain volume, compute the loading percentage, and provide real-time feedback to the driver. When the grain height is about to exceed the height of the front wall of the compartment, transition to the Overheight Warning status.
- Overheight Warning: Prompt the driver to move the vehicle forward until the grain height in the discharge port area returns to a safe range.
- Loading Complete: Once loading is complete, stop the discharge from the port and prompt the vehicle to leave the loading area. Standby Status: The system waits for the next vehicle to enter the loading area.

By implementing these steps, the system can accurately identify the current status of grain loading and provide appropriate instructions to ensure safe and efficient loading operations. The real-time feedback and automated instructions reduce manual intervention, improve loading efficiency, and enhance safety during the bulk grain-loading process.

## 4. Experiments

To evaluate the performance of the PNGL network in recognizing bulk grain-loading status, multiple experiments were conducted on the collected bulk grain-loading dataset.

### 4.1. Experimental Setup

To ensure training quality and increase model robustness, data augmentation techniques such as random jittering and random flipping were applied to part of the collected data. The hyperparameters for the model were set as follows: batch size of 16,200 epochs, initial learning rate of 0.001, learning rate decay of 0.5, with decay applied every 20 iterations, and using the AdamW optimizer. After 200 epochs of iterative training, the best segmentation model was used to segment the data in the test set.

### 4.2. Evaluation Metrics

The following evaluation metrics were used to quantitatively compare and analyze the classification and segmentation results of the bulk grain-loading point clouds: Overall Accuracy (OA), Mean Accuracy (mAcc) [26], and Mean Intersection over Union (mIoU) [27].

(8) $$\mathrm{OA}=\frac{\sum_{i=1}^{C}\left(TP_{i}+TN_{i}\right)}{\sum_{i=1}^{C}\left(TP_{i}+TN_{i}+FP_{i}+FN_{i}\right)},$$

(9) $$\mathrm{mAcc}=\frac{1}{C}\sum_{i=1}^{C}\frac{TP_{i}}{TP_{i}+FN_{i}},$$

(10) $$\mathrm{mIoU}=\frac{1}{C}\sum_{i=1}^{C}\frac{{TP}_{i}}{TP_{i}+FP_{i}+FN_{i}}$$

where *C* is the total number of classes, *TP_i_* is the number of true positives for class *i*, *TN_i_* is the number of true negatives for class *i*, *FP_i_* is the number of false positives for class *i*, *FN_i_* is the number of false negatives for class *i*.

### 4.3. Results of Experiments

**Figure 13:**
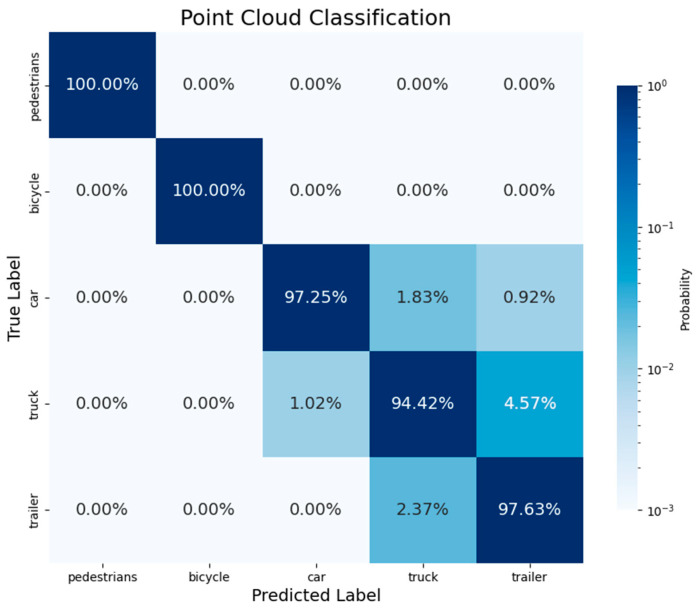
Point cloud classification.

To evaluate the point cloud-classification performance of our network, we calculated the prediction probabilities for point cloud classification and segmentation and plotted the corresponding heatmap, as shown in Figure 13. From the figure, it can be seen that for the point cloud-classification task, our method achieved an Overall Accuracy (OA) of 97.9%. Specifically, the classification results for pedestrian and bicycle point clouds were the best, mainly because pedestrian and bicycle targets are smaller and have significantly different features compared to vehicle point clouds, resulting in optimal classification performance. The classification accuracies for the two main targets, trucks and trailers, were 94.4% and 97.6%, respectively, generally meeting the task requirements. However, we observed some classification errors, which may be due to grain loaded inside the cargo area obstructing part of the vehicle features, leading to misclassification. To address this issue, we plan to add a verification procedure in the future to quickly check the classified point clouds and further improve accuracy.

**Figure 14:**
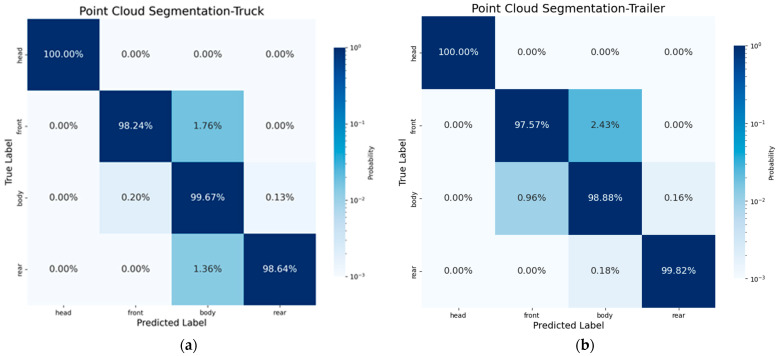
Point cloud segmentation. Head is cab of vehicle, front is front wall of cargo area, rear is rear wall of cargo area, body is cargo area. (**a**) the point cloud segmentation result of truck and (**b**) the point cloud segmentation result of trainer.

The point cloud segmentation predictions for the two research objects are shown in Figure 14a,b. For the segmentation of truck components, the overall performance accuracy is relatively good, with segmentation errors concentrated between the body and the front and rear walls. This is because the increased height of the grain pile covers the cargo walls, making them difficult to distinguish. However, the extraction of vehicle data is completed in the empty load status, so it only causes the grain point cloud extraction to be wider than it actually is. Therefore, the aforementioned phenomenon is within a reasonable range and does not affect the effectiveness of our method. Similarly, the trailer also has this issue, but because the rear half of the trailer is not loaded too high, the distinction between the trailer body and the rear wall is better.

### 4.4. Comparative Experiments

To fairly demonstrate the robustness and effectiveness of our architecture, we compared it with other representative point cloud models, including PointNet [9], PointNet++ [10], ShellNet [14], and PointMLP [15]. Additionally, we incorporated metrics for FLOPs (Floating Point Operations) and Params (Parameters) to assess the complexity and scale of the model’s parameters, ensuring that the model is practical and usable in engineering applications. The test results for the two tasks are shown in Table 3 and Table 4. 

In the point cloud-classification task, the PointNet network exhibited the lowest classification accuracy. This phenomenon is attributed to the fact that PointNet only extracts global features, neglecting the spatial adjacency relationships between points, which contain some key feature information. PointNet++, with its hierarchical structure, can extract local point information, thus achieving better point cloud-classification results. However, due to the similar geometric structures at the edges, feature aggregation through local neighborhoods can lead to overly smooth feature transitions. Moreover, when grain obstructs the vehicle walls, it causes edge sparsity and results in the loss of local details. Shellnet assigns ordered convolutional weights to the unordered point cloud by statistical features within concentric spherical shells, but loses local details and high-frequency information when aggregating features from each shell layer, leading to inferior performance in bulk grain-loading tasks compared to PointNet++. In contrast, PointMLP recursively aggregates local features through a feed-forward residual MLP network, demonstrating superior performance, but with higher FLOPs and Params, consuming more computational resources. Overall, our network, improved specifically for the characteristics of bulk grain loading, achieved an OA of 97.9% and a mAcc of 98.1% in the point cloud-classification task, offering high accuracy with a simple model, making it more suitable for practical engineering applications.

**Figure 15:**
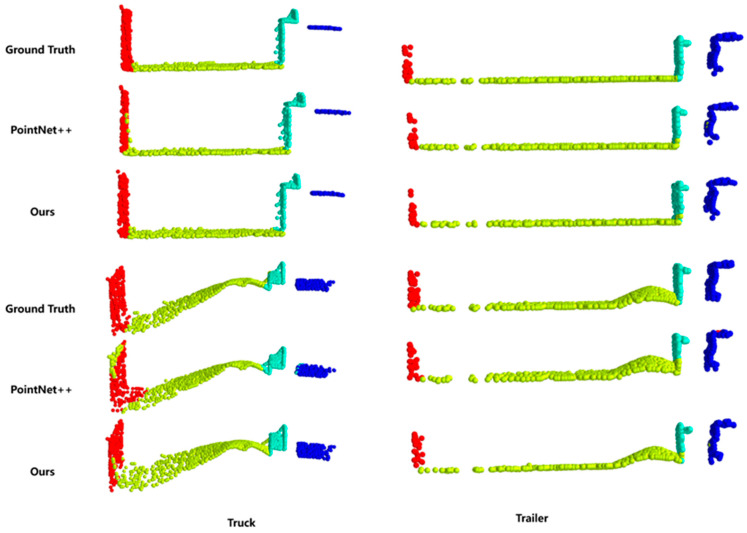
Segmentation results comparison. Blue represents the vehicle’s cab, cyan represents the front wall of the cargo area, green represents the cargo area itself, and red represents the rear wall of the cargo area.

In the point cloud-segmentation task, these models showed similar characteristics. Figure 15 shows the comparison of point cloud-segmentation results between this method and the PointNet++ network. For the PointNet++ network, it can be observed that there are classification errors at the edges of components and the junctions between the vehicle walls and the cargo area. These errors are mainly due to dust interference and the occlusion of vehicle components by grain, leading to similar structures or feature loss. For the proposed network in this paper, the results closely match the actual situation. It correctly classifies the cab, front wall, rear wall, and cargo area for both types of vehicles. Even when grain accumulates and obscures the cargo area walls, it correctly segments the corresponding components. This is primarily because the proposed method includes point cloud filtering, which reduces dust interference. It also uses octree sampling and a weighted cross-entropy loss function, which better preserves local details, reduces the weight of lost features, and addresses the classification imbalance caused by the characteristics of grain and environmental factors. This resulted in an OA of 99.1% and a mIOU of 96.6%. In summary, our method maintains high accuracy in target recognition and vehicle component segmentation during the bulk grain-loading process, making it more suitable for determining the bulk grain-loading status.

The above results indicate that this method maintains high accuracy for target recognition and vehicle component segmentation during the bulk grain-loading process, making it more suitable for determining the bulk grain-loading status.

## 5. Discussion

Currently, research on recognizing the bulk grain-loading status is still in its infancy. This paper utilizes LiDAR sensors for data collection and relies on deep learning networks to accurately detect and classify vehicles and their loading status, achieving the recognition of bulk grain-loading statuses and filling a gap in this field. 

Experimental results demonstrate that the proposed PNGL network has achieved high accuracy in both point cloud-classification and component-segmentation tasks. To provide a comprehensive assessment, our results were compared with several state-of-the-art deep learning methods for point clouds, including PointNet [9], PointNet++ [10], Shellnet [14], and PointMLP [15]. In the point cloud-classification task, our PNGL network achieved an OA of 97.9% and a mAcc of 98.1%. By effectively capturing both global and local features, it surpassed these advanced methods even in the challenging environment of bulk grain-loading tasks characterized by occlusions and dynamic point clouds. In the component-segmentation task, it achieved a segmentation accuracy of 99.1% OA and 96.6% mIOU. It demonstrated excellent segmentation performance, especially in differentiating components in close proximity and handling partial occlusions caused by grains and dust, while maintaining a lightweight structure that reduces computational resource consumption, making it more suitable for bulk grain-loading tasks. 

This study is dedicated to solving the problems of high labor intensity, poor safety, and low loading efficiency in the bulk grain-loading process. Due to the particularity of bulk grain-loading tasks, occlusions caused by loading and unloading equipment and vehicle body components leading to incomplete point clouds, as well as interference from dust, pose challenges to the task of recognizing grain-loading status. Our study addresses several key issues related to the bulk grain-loading process:

- How does the PNGL network handle occlusions and dust interference compared to other methods? The PNGL network, combined with octree sampling and advanced feature propagation techniques, enhances its ability to filter out noise and maintain high precision even in dusty and occluded environments. The improvement in performance metrics compared to other methods proves this point.
- What are the advantages of using the PNGL network in dynamic and sparse point cloud scenarios? The dynamic nature of grain loading presents challenges for point cloud processing. The hierarchical feature extraction and global average pooling of the PNGL network reduce sensitivity to sparsity and motion in point clouds, thereby achieving more reliable classification and segmentation results.
- Why is the PNGL network more suitable for bulk grain-loading applications? The bulk grain-loading process requires precise detection of load levels to prevent overflow and optimize space utilization. The PNGL network can accurately segment vehicle components and detect grain shapes, ensuring that it can provide real-time feedback for efficient and safe loading operations.

We address research questions by associating the deep learning network with the initial goals of improving loading efficiency, reducing labor intensity, and assisting in the automation of loading vehicles. Specifically, the proposed method effectively detects and segments common transport vehicle types, including the cab, front and rear walls of the cargo area, the interior of the cargo area, and the grain. By analyzing the interior of the cargo area, the method recognizes the grain shape, and by assessing the relative positions of vehicle components, it detects the vehicle’s position. Compared to traditional manual detection and other deep learning models, this method has the following advantages:

- Reliable Loading Status Recognition: By comparing the detected grain shape and height with predefined thresholds and setting buffer zones, the method ensures that all four vehicle statuses can be correctly determined. This prevents accidents caused by grain height exceeding the cargo area’s limits due to occasional recognition errors.
- Intelligent Prompting: Based on the detected status and grain shape, the system outputs vehicle movement instructions to the driver, effectively guiding the loading process. This significantly reduces the labor intensity of workers, improves safety, and enhances loading efficiency.
- Higher Recognition Accuracy: Compared to commonly used deep learning models, this method shows significant improvements in accuracy for both point cloud-classification and -segmentation tasks.
- Robustness in Complex Environments: This method does not require large-scale modifications to the loading site. It can accurately detect vehicle positions and recognize loading statuses even in the presence of dust interference and occlusion in the point cloud data.

## 6. Conclusions

This paper addresses the issues present in traditional bulk grain loading and proposes a LiDAR-based method for recognizing bulk grain-loading status. This method involves placing two LiDAR sensors in the loading area to acquire point cloud data. Through data fusion and preprocessing, a complete vehicle point cloud is obtained. Based on the characteristics of the bulk grain-loading process, a deep learning network architecture, PNGL, is designed for vehicle recognition and component segmentation. This enables the detection of vehicle positions, perception of grain shapes, and recognition of bulk grain-loading status. Finally, the method outputs prompt instructions based on the recognized grain-loading status, effectively solving the communication difficulties, safety risks, and inefficiencies present in traditional bulk grain-loading processes.

In future research, we plan to further streamline the model structure to ensure performance while reducing hardware dependency and costs. We aim to collect data from a wider variety of grain-loading scenarios to enrich the dataset, enhance the model’s generalization ability, robustness, and stability, and further promote the development and application of automated bulk grain-loading technology.

## Figures and Tables

**Table 1:** LiDAR parameters.

Parameter	Value
Measurement Principle	Time of Flight
Detection Range	200 m
Measurement Accuracy	±3 cm
Scanning Frequency	10 Hz
Horizontal Field of View	360°
Vertical Field of View	−15° to 15°
Angular Resolution	0.18° (horizontal), 2° (vertical)
Dimensions	102 mm (diameter) × 81 mm (height)

**Table 2:** Bulk grain-loading dataset.

Categories	Pedestrians	Cars	Bicycles	Trucks	Trailers
Classification training	125	108	22	186	864
Classification testing	41	36	8	62	288
Segmentation training	—	—	—	180	569
segmentation testing	—	—	—	60	261

**Table 3:** Point cloud-classification task.

Methods	OA/%	mAcc/%	FLOPs/G	Params/M
PointNet [9]	91.7	88.3	0.4	3.5
PointNet++ [10]	95.8	95.4	4.0	1.7
ShellNet [14]	93.4	90.7	0.3	0.5
PointMLP [15]	98.8	98.3	31.8	13.3
Our	97.9	98.1	2.1	1.7

**Table 4:** Point cloud-segmentation task.

Methods	OA/%	mIOU/%	Truck IOU/%	Trailer IOU/%	FLOPs/G	Params/M
PointNet [9]	91.0	87.0	97.4	84.6	4.9	3.6
PointNet++ [10]	95.9	94.3	97.8	93.0	4.9	1.0
ShellNet [14]	92.8	91.0	95.1	89.6	1.0	0.9
PointMLP [15]	98.3	96.5	98.4	95.8	35.3	16.7
Our	99.1	96.6	98.9	95.9	4.2	1.0

## Data Availability

The data are not publicly available due to restrictions on privacy.

## References

[1] Mahima K.T.Y., Perera A., Anavatti S., Garratt M. (2023). Exploring Adversarial Robustness of LiDAR Semantic Segmentation in Autonomous Driving. Sensors.

[2] Ghasemieh A., Kashef R. (2022). 3D object detection for autonomous driving: Methods, models, sensors, data, and challenges. Transp. Eng..

[3] Soori M., Arezoo B., Dastres R. (2023). Artificial intelligence, machine learning and deep learning in advanced robotics, a review. Cogn. Robot..

[4] Qian R., Lai X., Li X. (2022). 3D object detection for autonomous driving: A survey. Pattern Recognit..

[5] Mao J., Shi S., Wang X., Li H. (2023). 3D object detection for autonomous driving: A comprehensive survey. Int. J. Comput. Vis..

[6] Wang Z., Wu Y., Niu Q. (2019). Multi-sensor fusion in automated driving: A survey. IEEE Access.

[7] Wang B., Zhu M., Lu Y., Wang J., Gao W., Wei H. (2021). Real-time 3D object detection from point cloud through foreground segmentation. IEEE Access.

[8] Ruan X., Liu B. (2020). Review of 3d point cloud data segmentation methods. Int. J. Adv. Netw. Monit. Control..

[9] Qi C.R., Su H., Mo K., Guibas L.J. Pointnet: Deep learning on point sets for 3D classification and segmentation. Proceedings of the 2017 IEEE Conference on Computer Vision and Pattern Recognition (CVPR 2017).

[10] Qi C.R., Yi L., Su H., Guibas L.J. Pointnet++: Deep hierarchical feature learning on point sets in a metric space. Proceedings of the Neural Information Processing Systems (NIPS 2017).

[11] Qian G., Li Y., Peng H., Mai J., Hammoud H., Elhoseiny M., Ghanem B. (2022). Pointnext: Revisiting pointnet++ with improved training and scaling strategies. Adv. Neural Inf. Process. Syst..

[12] Wang Y., Sun Y., Liu Z., Sarma S.E., Bronstein M.M., Solomon J.M. (2019). Dynamic graph cnn for learning on point clouds. ACM Trans. Graph. (Tog).

[13] Zhang K., Hao M., Wang J., de Silva C.W., Fu C. (2019). Linked dynamic graph cnn: Learning on point cloud via linking hierarchical features. arXiv.

[14] Zhang Z., Hua B.S., Yeung S.K. Shellnet: Efficient point cloud convolutional neural networks using concentric shells statistics. Proceedings of the IEEE/CVF International Conference on Computer Vision.

[15] Ma X., Qin C., You H., Ran H., Fu Y. (2022). Rethinking network design and local geometry in point cloud: A simple residual MLP framework. arXiv.

[16] Bello S.A., Yu S., Wang C., Adam J.M., Li J. (2020). Deep learning on 3D point clouds. Remote Sens..

[17] Zhang R., Zhang G., Yin J., Jia X., Mian A. (2023). Mesh-based DGCNN: Semantic Segmentation of Textured 3D Urban Scenes. IEEE Trans. Geosci. Remote Sens..

[18] Gamal A., Wibisono A., Wicaksono S.B., Abyan M.A., Hamid N., Wisesa H.A., Jatmiko W., Ardhianto R. (2020). Automatic LIDAR building segmentation based on DGCNN and euclidean clustering. J. Big Data.

[19] Liu Y., Li W., Liu J., Chen H., Yuan Y. (2023). GRAB-Net: Graph-based boundary-aware network for medical point cloud segmentation. IEEE Trans. Med. Imaging.

[20] Camuffo E., Mari D., Milani S. (2022). Recent advancements in learning algorithms for point clouds: An updated overview. Sensors.

[21] Dang X., Jin P., Hao Z., Ke W., Deng H., Wang L. (2023). Human Movement Recognition Based on 3D Point Cloud Spatiotemporal Information from Millimeter-Wave Radar. Sensors.

[22] Hao H., Jincheng Y., Ling Y., Gengyuan C., Sumin Z., Huan Z. (2023). An improved PointNet++ point cloud segmentation model applied to automatic measurement method of pig body size. Comput. Electron. Agric..

[23] Horaud R., Hansard M., Evangelidis G., Ménier C. (2016). An overview of depth cameras and range scanners based on time-of-flight technologies. Mach. Vis. Appl..

[24] Li Y., Ibanez-Guzman J. (2020). Lidar for autonomous driving: The principles, challenges, and trends for automotive lidar and perception systems. IEEE Signal Process. Mag..

[25] Wen X., Hu J., Chen H., Huang S., Hu H., Zhang H. (2023). Research on an adaptive method for the angle calibration of roadside LiDAR point clouds. Sensors.

[26] Long J., Shelhamer E., Darrell T. (2015). Fully convolutional networks for semantic segmentation. IEEE Trans. Pattern Anal. Mach. Intell..

[27] Turpin A., Scholer F. User performance versus precision measures for simple search tasks. Proceedings of the 29th Annual International ACM SIGIR Conference on Research and Development in Information Retrieval.

